# Genetic Biomarkers for Statin-Induced Myopathy

**DOI:** 10.3390/ijms262211144

**Published:** 2025-11-18

**Authors:** Diana Prieto-Peña, Juan David Urriago-Gil, Gonzalo Ocejo-Vinyals, Carmen García-Ibarbia, Zaida Salmon-González, Marta Martin-Millán, Cristina Corrales-Selaya, Verónica Pulito-Cueto, Raquel López-Mejías, Ricardo Blanco, Jose Luis Hernandez

**Affiliations:** 1 Department of Rheumatology, Hospital Universitario Marqués De Valdecilla, IDIVAL, 39008 Santander, Spain; diana.prieto.pena@gmail.com (D.P.-P.); j.urreuma23@gmail.com (J.D.U.-G.);; 2 Immunopathology Research Group, IDIVAL Health Research Institute of Cantabria, 39008 Santander, Spainrlopez@idival.org (R.L.-M.); 3 Department of Immunology, Hospital Universitario Marqués De Valdecilla, IDIVAL, 39008 Santander, Spain; 4 Department of Internal Medicine, Hospital Universitario Marqués De Valdecilla, IDIVAL, 39008 Santander, Spain; 5 Medicine Department, University of Cantabria, 39011 Santander, Spain

**Keywords:** genetics, statins, myopathy necrotizing myopathy, HLA-DRB1*11, *SLCO1B1*, myopathy

## Abstract

Statin exposure has been associated with a broad spectrum of muscle toxicity, ranging from asymptomatic creatine kinase (CK) elevation to immune-mediated necrotizing myopathy (IMNM) with anti-3-hydroxy-3-methylglutaryl-coenzyme A reductase (HMGCR) antibodies. The mechanisms underlying these adverse effects are not fully understood, and genetic predisposition may play a role. This observational study evaluated the association of HLA-DRB1*11 and *SLCO1B1 rs4149056* variants with statin-induced muscle toxicity. A total of 62 statin-exposed patients treated at a single tertiary center were included and classified as follows: IMNM with anti-HMGCR antibodies (n = 11), non-immune myotoxicity (n = 20), and statin-exposed controls without myopathy (n = 31). The mean age was 66 ± 7.5 years, and 62% were women. The frequency of the HLA-DRB1*11 allele was significantly higher in patients with anti-HMGCR IMNM compared to those with non-immune myotoxicity (81.0% vs. 25.0%; OR = 13.5, 95% CI 1.73–15.3; *p* < 0.01) and controls (81.0% vs. 17.2%; OR = 21.6, 95% CI 2.87–23.7; *p* < 0.01). No significant difference was found between the non-immune myotoxicity and control groups. Likewise, the *SLCO1B1 rs4149056* variant showed no association with either IMNM or non-immune muscle toxicity. These findings confirm a strong association between the HLA-DRB1*11 allele and anti-HMGCR IMNM. This genetic marker may help to better distinguish immune-mediated from non-immune forms of statin-related myopathy.

## 1. Introduction

The clinical spectrum of statin-related myopathies varies widely from asymptomatic creatinine kinase (CK) elevations, pain, or muscle weakness to severe forms of necrotizing immune-mediated myopathy by anti-3-hydroxy-3-methylglutaryl coenzyme A reductase (HMGCR) antibodies [1]. The prescription of statins has increased progressively in the last decade. They act through competitive, partial, and reversible inhibition of the HMGCR enzyme that catalyzes the conversion of HMG-CoA to mevalonate, which is a key metabolite in cholesterol biosynthesis [2].

Approximately 10–30% of patients receiving statin therapy experience adverse effects, predominantly muscle-related toxicity, most commonly of mild severity [3]. The principal pathophysiological mechanisms implicated include a reduction in the synthesis of isoprenylated proteins, leading to decreased intracellular markers, particularly RhoC protein, which results in inhibition of smooth muscle cell proliferation and induction of myocyte apoptosis. Statins have been associated with dysregulation of the ubiquitin-proteasome system and depletion of coenzyme Q10, culminating in impaired mitochondrial respiratory chain function. Furthermore, statins exert an immunomodulatory effect through upregulation of major histocompatibility complex class I (MHC-I) expression on the sarcolemmal membrane, thereby promoting myocyte apoptosis [3,4].

A subset of patients on statin therapy develop a distinct form of myopathy characterized by an immune-mediated response involving anti-HMGCR antibodies. Anti-HMGCR immune-mediated necrotizing myopathy (IMNM) is classified as a subtype of inflammatory myopathy with a severe clinical course. In contrast to conventional statin-associated myotoxicity, affected patients exhibit persistent clinical symptoms despite statin withdrawal and frequently require immunosuppressive therapy [5]. Although the precise etiopathogenesis remains incompletely elucidated, the prevailing hypothesis suggests that anti-HMGCR antibodies target HMGCR antigens expressed on the sarcolemmal membrane, triggering muscle fiber necrosis via complement pathway activation [5].

Demographic, comorbid, and pharmacological aspects have been implicated as predisposing factors for the development of statin-associated myotoxicity. Among these, advanced age, hypothyroidism, vitamin D deficiency, high statin dosages, and potential drug–drug interactions have been specifically reported [4]. Nonetheless, the reasons underlying the variability in clinical presentation, ranging from mild asymptomatic increases in creatine kinase (CK) to severe muscle toxicity, remain poorly understood. Genetic predisposition has been proposed as a potential explanatory factor. Specifically, variants in the *SLCO1B1* gene have been associated with an increased susceptibility to severe statin-induced myotoxicity [6]. Moreover, a recent study conducted at our institution supported previous findings by Mammen et al. [7], demonstrating a significant association between the HLA-DRB1*11 allele and a high risk of developing anti-HMGCR immune-mediated necrotizing myopathy (IMNM) [8]. These findings support the hypothesis that genetic factors may play a critical role in the etiopathogenesis of statin-induced myopathies; however, it remains unclear whether these genetic markers are phenotype-specific.

In light of these considerations, the present study aimed to evaluate whether genetic variants in *SLCO1B1* and the HLA-DRB1*11 allele can be predictive biomarkers to identify patients at elevated risk for severe myopathy, thereby informing clinical decision-making to avoid statin use in susceptible individuals and guiding optimal therapeutic strategies.

## 2. Results

### 2.1. Study Population Assessment

The overall population comprised 62 patients, distributed across three groups: IMNM (n = 11), non-immune myotoxicity (n = 20), and controls (n = 31). The mean age across the three groups was 66 ± 7.5 years, and the majority of patients were female (62%).

Comparative analyses were performed among all three groups; however, for clarity, only the comparison between Group 1 and Group 2 is presented in Table 1 and Table 2. The most relevant and statistically meaningful findings from these analyses are summarized below.

Compared to patients with non-immune myotoxicity, the frequency of myalgia, muscle weakness, elevated serum CK levels, and dysphagia was higher in anti-HMGCR IMNM patients. Atorvastatin was the most frequently used statin, with a significant difference observed in anti-HMGCR IMNM patients compared to the other groups; however, this may be attributed to the small sample size in this group. No statistically significant differences were observed in the duration of statin exposure among the three groups; nevertheless, the anti-HMGCR group exhibited greater exposure. Additional clinical characteristics are shown in Table 1.

### 2.2. Genetic Assessment

The frequency of the HLA-DRB1*11 phenotype was significantly increased in HMGCR-IMNM patients compared to patients with non-immune myotoxicity (81.0% vs. 25.0%; OR = 13.5 CI: 1.73–153.21 *p* < 0.01) and controls (81.0% vs. 17.2%, OR = 23.4 CI: 3.13–256.11 *p* < 0.01). In contrast, no significant association was found between patients with non-immune myotoxicity and controls (Table 2 and Table 3).

In the allelic analysis, HMGCR-IMNM patients showed a stronger association with the HLA-DRB1*11:01 allele compared to non-immune myotoxicity and controls (OR = 24.0 CI: 2.57–293.86; *p* < 0.01 and OR = 1.73 CI: 0.33–8.84; *p* = 0.4, respectively). No differences were observed for the HLA-DRB1*11:02 and HLA-DRB1*11:04 alleles across all groups (Table 3).

When analyzing *SLCO1B1* genotypes and alleles, no association was found with any of the three groups (Table 2 and Table 4).

## 3. Discussion

IMNMs are a group of diseases characterized by myofibrillar necrosis, muscle weakness, and raised serum CK levels [9]. The identification of the anti-HMGCR autoantibody (initially termed anti-200kd/100kd) in 2010 led to a better understanding of the pathophysiological mechanisms of the disease, as well as the characterization of the clinical phenotype and prognosis of affected patients [10]. The HMGCR enzyme catalyzes the conversion of HMG-CoA to mevalonic acid, a critical step in cholesterol synthesis [11]. This pathway is inhibited by statins, medications widely used in clinical practice, due to their benefits in cardiovascular disease [12]. Additionally, up to 20% of patients exposed to statins develop muscle symptoms or elevated serum CK levels, a condition known as statin-induced non-immune muscle toxicity [13]. In the original description, statin exposure was reported in 63% of patients with anti-HMGCR positivity [10]. Nonetheless, heterogeneous rates of prior exposure to these pharmacological agents have been reported across studies conducted worldwide [14,15], suggesting the influence of factors such as genetics, age, and duration of drug exposure [16].

The identification of genes within the major histocompatibility complex has demonstrated an association between genetic factors and the development of the disease [17], with certain alleles found to be more prevalent in patients with IMNM compared to the general population [18]. Additionally, the *SLCO1B1* gene encodes the OATP1B1 protein, which is responsible for the hepatic uptake of statins, and its identification has garnered interest in the study of the pharmacogenomics of these lipid-lowering medications [19].

Our study analyzed the association of the HLA-DRB1*11 and *SLCO1B1* genes in three groups of patients exposed to statins, with or without the development of non-immune muscle toxicity or anti-HMGCR IMNM. The clinical characteristics of patients with anti-HMGCR positivity were consistent with those reported by several authors in other geographical areas [15,16,17,18,19,20]. The frequency of the HLA-DRB1*11 gene was significantly higher in the anti-HMGCR group compared to the other two groups. These findings are consistent with reports from other authors, where HLA-DRB1*11 positivity has been documented in 60–70% of statin-exposed patients with anti-HMGCR IMNM [21], as well as in patients without prior statin exposure [22]. One hypothesis explaining these findings is the antigenic presentation of enzyme peptides by this major histocompatibility complex to effector cells of the immune system [23]. Regarding the allelic analysis, our study identified a significant association only with the HLA-DRB1*11:01 allele, in the anti-HMGCR IMNM group, with no significant associations observed for the other alleles studied. Allelic variations in the HLA-DRB1*11 gene have shown different associations in this patient group, such as HLA-DRB1*08:03 in patients with prior statin use and HLA-DRB1*11:01 in patients with paraneoplastic disease or coexisting connective tissue disorders [24].

In the group of patients with non-immune muscle toxicity, no statistical association was found with the HLA-DRB1*11 gene. Muscle toxicity has been reported in up to 20% of patients receiving statins [25]. Factors such as age over 80 years, untreated hypothyroidism, low serum vitamin D levels, hypertension, high statin doses, and concomitant use of medications affecting cytochrome P450 systems (CYP3A4, CYP2C9) and the OATP1B1 protein have been associated with an increased incidence of this adverse effect of statins [1]. In this context, genetic mutations in cytochrome isoenzymes (particularly CYP3A4), polymorphisms in CoQ2 (responsible for coenzyme Q10 synthesis), and genes encoding glycine amidinotransferase (GATM) have been proposed as potential contributors to statin-induced muscle toxicity. However, current evidence supporting routine genetic testing for these alterations remains limited [26,27].

Our study could not demonstrate an association between different *SLCO1B1* genotypes and the groups with non-immune muscle toxicity or anti-HMGCR IMNM. The rs4149056 variant of *SLCO1B1* has been identified as the most robust genetic alteration associated with non-immune muscle toxicity in patients exposed to statins [28]. However, the operational performance of detecting this gene varies; based on published data, the sensitivity and specificity of the test are 70.4% and 73.7%, respectively, with corresponding positive and negative predictive values of 4.1% and 99.4%, respectively [29,30]. A recent network meta-analysis identified a moderate association between the rs4149056 polymorphism of the *SLCO1B1* gene and statin-induced non-immune muscle toxicity (OR = 2.1; 95% CI: 1.7–2.6), with intraclass differences in genetic predictive risks among statins [31]. Nevertheless, the generalization of these findings requires further studies for implementation in routine clinical practice.

These findings provide insights into the role of HLA-DRB1*11 genetic testing in differentiating non-immune muscle toxicity from anti-HMGCR IMNM in patients receiving statins, particularly in settings where autoantibody testing for inflammatory myopathies, skeletal muscle biopsy is unavailable, or conclusive diagnostic studies are obtained. Unlike reports from other authors, our study could not demonstrate an association between the rs4149056 variant of *SLCO1B1* and non-immune-mediated muscle toxicity. This may be attributed to study limitations, primarily the small sample size, which reduces statistical power and increases the risk of obtaining non-representative results. Additionally, the influence of environmental factors and other uncontrolled exposures may contribute to gene expression and affect the occurrence of the outcome (effect-modifying or interacting variables). In the future, identifying new genetic variants and their influence on the development of non-immune muscle toxicity or anti-HMGCR IMNM in patients exposed to statins will enhance diagnostic accuracy for both conditions and facilitate the early identification of patients at risk of muscle damage following exposure to these lipid-lowering agents.

The limited sample size is a relevant constraint of this study and may influence the strength and generalizability of the conclusions. This reflects the low prevalence of anti-HMGCR IMNM and the single-center design of our work. Collaborative multicenter studies involving larger cohorts will be essential to confirm these findings and provide broader insights into the genetic background of this rare disease.

## 4. Materials and Methods

We conducted an observational study that included all patients diagnosed with anti-HMGCR IMNM (Group 1) who attended a single tertiary referral center, the University Hospital Marqués de Valdecilla, in Cantabria (Northern Spain), from January 2016 to December 2023. Patients with IMNM where characterized by: (1) acute or subacute onset of symmetrical proximal muscle weakness, (2) elevated CK levels, and (3) positivity for anti-HMG-CoAR antibodies.

Then, we recruited two comparative groups of patients: patients with non-immune myotoxicity (Group 2) and controls (Group 3). Group 2 comprised patients with non-immune muscle toxicity, as defined by an expert panel [1] (see Supplementary Materials). Group 3 included patients on statin therapy for at least one year without developing myalgia, weakness, or elevated serum CK levels.

Patients diagnosed with dermatomyositis, antisynthetase syndrome, overlap syndrome myositis, inclusion body myositis, muscular dystrophy, metabolic myopathy, myopathy induced by other medications, or elevated serum CK levels before statin initiation and due to other causes (e.g., exercise) were excluded.

A review of the medical records of the included patients was conducted, and variables of interest were collected in a standardized and anonymized database. Peripheral blood samples were obtained from all patient groups, and DNA extraction was performed using the Promega Biotech Ibérica, S.L. system (Madrid, Spain). DNA samples were stored at −20 °C until analysis.

### 4.1. Genetic Analysis

High-resolution genotyping of the HLA-DRB1*11 gene was conducted using the NGSgo-AmpX v2 amplification primer (Genome Diagnostics B.V., Utrecht, The Netherlands) through next-generation sequencing, with data analyzed using NGSengine software (GenDx, Utrecht, The Netherlands; available at https://www.gendx.com/products/ngsengine/; accessed on 1 June 2024). For the identification of the rs4149056 polymorphism in the *SLCO1B1* gene, TaqMan probes with real-time PCR were employed on the QuantStudio^TM^ 7 Flex real-time polymerase chain reaction system (Applied Biosystems, Foster City, CA, USA). Allelic frequency differences were analyzed for both genes. Informed consent was obtained from patients at the time of sample collection. The study was approved by the Ethics Committee of Cantabria (2023.286, 22 September 2023).

### 4.2. Statistical Analysis

The association between the evaluated genes of the three groups was assessed using odds ratios (OR) with 95% confidence intervals (CI). Matching with anti-HMGCR IMNM patients was performed for the two comparative groups as follows: 2:1 for patients with non-immune muscle toxicity and 3:1 for controls. Logistic regression was conducted to adjust for potential confounding factors. Differences in allelic frequencies of each genetic biomarker were analyzed concerning categorical variables in the population using OR, 95% CI, and Fisher’s exact test. To compare quantitative variables with normal distribution, Student’s *t*-test was used, while the Mann–Whitney U test was applied for non-normally distributed variables. The Wilcoxon signed-rank test was used for comparing related samples. Statistical significance was set at a *p*-value < 0.05 for all the calculations. Data analyses were performed using IBM SPSS Statistics for Windows, Version 28.0. Armonk, NY, USA: IBM Corp.

## 5. Conclusions

In conclusion, in patients exposed to statins, the presence of the HLA-DRB1*11 gene was significantly associated with anti-HMGCR-IMNM disease compared to those with non-immune muscle toxicity. This finding might serve as a valuable tool for more accurately differentiating both conditions. Conversely, no association was found between the rs4149056 variant of *SLCO1B1* gene and the presence of anti-HMGCR-IMNM or non-immune muscle toxicity.

## Figures and Tables

**Table 1:** Demographic, clinical features and laboratory parameters in patients with anti-HMGCR IMNM, non-immune-mediated statin myotoxicity and patients taking statins without myotoxicity (controls).

Variables	Group1 Anti-HMGCR IMNM Patients (n = 11)	Group 2 Non-Immune Myotoxicity (n = 20)	Group 3 Controls (n = 31)	*p* (Group 1 vs. Group 2)
Demographic features				
Age at diagnosis (years), mean ± SD	66.7 ± 7.9	58.3 ± 9.5	67.0 ± 10.6	0.018
Sex (women), n (%)	6 (54.5)	10 (50)	21 (72.4)	0.81
Current and former smokers, n (%)	5 (45.5)	13 (65)	10 (34.5)	0.29
Hypertension, n (%)	9 (81.8)	8 (40)	14 (48.3)	0.03
Type 2 Diabetes mellitus, n (%)	9 (81.8)	0 (0)	2 (6.8)	<0.01
Previous diagnosis of hypothyroidism requiring hormone replacement, n (%)	4 (36.4)	3 (15)	3 (10.3)	0.21
Statin exposure				
Number of received statins, median [IQR]	1 [1–2]	2 [1–2]	1 [1–1]	0.23
Type of statin,				
- Atorvastatin, n (%)	11 (100)	12 (60)	20 (68.9)	0.01
- Rosuvastatin, n (%)	2 (18.2)	9 (45)	5 (17.2)	0.14
- Simvastatin, n (%)	1 (9.1)	6 (30)	7 (24.1)	0.18
- Pitavastatin, n (%)	1 (9.1)	7 (35)	3 (10.3)	0.12
Time receiving statins (months), median [IQR]	72 [24–84]	54 [22–96]	60 [36–84]	0.92
Clinical manifestations				
Duration of symptoms after diagnosis (months), mean ± SD	4 [3–6]	3.5 [2–8]	-	0.65
Myalgias, n (%)	10 (90.9)	11 (55)	0 (0)	0.04
Muscle weakness	11 (100)	4 (20)	0 (0)	<0.01
Proximal lower limb predominance, n (%)	9 (81.8)	0 (0)	0 (0)	<0.01
Neck weakness, n (%)	2 (25)	0 (0)	0 (0)	0.05
Dysphagia, n (%)	2 (25)	0 (0)	0 (0)	0.05
Laboratory parameters				
Elevated CK, n (%)	11 (100)	17 (85)	0 (0)	0.54
CK (IU/L), median [IQR]	4276 [2294–9271]	494 [214–828]	-	<0.01
LDH (mg/dL), median [IQR]	466 [375–876]	216 [194–314]	-	<0.01
AST (mg/dL), median [IQR]	122 [88–272]	38 [27–66]	-	<0.01
ALT (mg/dL), median [IQR]	160 [144–377]	41 [27–56]	-	<0.01
CRP (mg/dL), median [IQR]	0.8 [0.6–3.0]	0.2 [0.2–0.6]	-	<0.01
ESR (mm/1st hour), median [IQR]	27.0 [12–60]	18 [12–20]	-	0.09
Low vitamin D levels, n/N (%)	9/9 (100)	12/16 (75)	2 (6.9)	0.26
Elevated TSH, n (%)	4 (36)	3 (15)	0 (0)	0.17

ALT: alanine aminotransferase; AST: aspartate aminotransferase; CK: creatinine kinase; CRP: C-reactive protein; ESR: erythrocyte sedimentation rate; IMNM: immune-mediated necrotizing myopathy; IQR: interquartile range; LDH: lactate dehydrogenase; SD: standard deviation; TSH: thyroid-stimulating hormone.

**Table 2:** Frequency of distribution of both genes and haplotypes in the three groups.

Variables	Group 1 Anti-HMGCR IMNM Patients (n = 11)	Group 2 Non-Immune Myotoxicity (n = 20)	Group 3 Controls (n = 29)	*p* *
HLA-DRB1*11 Allele Frequencies				
HLA-DRB1*11, n (%)	9 (81.8)	5 (25)	5 (17.2)	<0.01
*11:01, n (%)	8 (72.7)	2 (10)	3 (10.3)	<0.01
*11:02, n (%)	0 (0)	1 (5)	1 (3.5)	0.73
*11:04, n (%)	1 (9.1)	2 (10)	1 (3.5)	0.90
*SLCO1B1 rs4149056*				
TT	9 (81.8)	11 (55)	20 (64.5)	-
TC	2 (18.2)	7 (35)	11 (35.5)	0.24
CC	0 (0)	2 (10)	0 (0)	-
T	20 (90.9)	29 (72.5)	51 (82.3)	-
C	2 (9.1)	11 (27.5)	11 (17.7)	0.09

* *p* values are shown for the comparison between anti-HMGCR-IMNM patients and non-immune mediated myotoxicity patients.

**Table 3:** Association between HLA-DRB1*11 allele frequencies with non-immune-mediated statin myotoxicity, anti-HMGCR IMNM patients, and controls.

	Patients with Non-Immune Mediated Myotoxicity vs. Controls		Patients with Anti-HMGCR IMNM vs. Controls		Patients with Anti-HMGCR IMNM vs. Patients with Non-Immune Myotoxicity	
	OR [95% CI]	*p*	OR [95% CI]	*p*	OR [95% CI]	*p*
HLA-DRB1*11	1.73 [0.33–8.84]	0.44	23.4 [3.13–256.11]	<0.01	13.5 [1.73–153.21]	<0.01
*11:01	1.04 [0.08–10.00]	0.97	24.89 [3.27–213.99]	<0.01	24.0 [2.57–293.86]	<0.01
*11:02	1.58 [0.02–128.00]	0.75	-	0.56	-	0.46
*11:04	3.33 [0.16–203.06]	0.32	3.00 [0.03–243.15]	0.43	0.90 [0.014–19.48]	0.93

Values correspond to unadjusted ORs. OR: odds ratio; CI: confidence Interval.

**Table 4:** Association between *SLCO1B1 rs4149056* genotype frequencies with non-immune-mediated statin myotoxicity, anti-HMGCR IMNM patients, and controls.

	Patients with Myotoxicity vs. Controls		Patients with Anti-HMGCR IMNM vs. Controls		Patients with Anti-HMGCR IMNM vs. Patients with Myotoxicity	
	OR [95% CI]	*p*	OR [95% CI]	*p*	OR [95% CI]	*p*
TT	Ref.	-	Ref.	-	Ref.	-
TC	1.16 [0.2–4.4]	0.81	0.4 [0.04–2.5]	0.29	2.86 [0.39–33.8]	0.24
CC	-	0.07	-	-	-	0.22
T	Ref.	-	Ref.	-	Ref.	-
C	1.76 [0.60–5.0]	0.24	0.46 [0.05–2.4]	0.34	3.79 [0.69–38]	0.09

Values correspond to unadjusted ORs. OR: odds ratio; CI: confidence Interval.

## Data Availability

The original contributions presented in this study are included in the article/Supplementary Materials. Further inquiries can be directed to the corresponding authors.

## Supplementary Materials

The following supporting information can be downloaded at: https://www.mdpi.com/article/10.3390/ijms262211144/s1.
