# Supplementary material for: Genetic Biomarkers for Statin-Induced Myopathy
Source: Int J Mol Sci. 2025 Nov 18;26(22):11144. doi: 10.3390/ijms262211144 (PMC12653957; doi:10.3390/ijms262211144)
Supplement: Supplementary file 1 [file ijms-26-11144-s001.zip › ijms-3941410-supplementary.pdf]

Standardization for Statin-InducedMyotoxicity according to A Alfirevic et al.

Subtype 0: Mild elevation of creatine kinase (CK) levels (< 4 times the upper limit of normal, ULN) without clinical muscle symptoms.
Subtype 1: Presence of muscle symptoms (e.g., myalgia, weakness) without elevation of CK levels.
Subtype 2: Muscle symptoms accompanied by mild CK elevation (< 4 times ULN).
Subtype 3: Moderate CK elevation (> 4 times ULN and < 10 times ULN), with or without muscle symptoms.
Subtype 4: Significant CK elevation (> 10 times ULN and < 50 times ULN) with muscle symptoms.
Subtype 5: Severe CK elevation (> 10 times ULN with evidence of renal failure and muscle symptoms, or > 50 times ULN).
